# Nano-Architectural Approaches for Improved Intracortical Interface Technologies

**DOI:** 10.3389/fnins.2018.00456

**Published:** 2018-07-17

**Authors:** Youjoung Kim, Seth M. Meade, Keying Chen, He Feng, Jacob Rayyan, Allison Hess-Dunning, Evon S. Ereifej

**Affiliations:** ^1^Department of Biomedical Engineering, Case Western Reserve University, Cleveland, OH, United States; ^2^Advanced Platform Technology Center, Louis Stokes Cleveland Department of Veterans Affairs Medical Center, Cleveland, OH, United States

**Keywords:** nano-architecture, topography, intracortical microelectrodes, neuroinflammation, mechanotransduction

## Abstract

Intracortical microelectrodes (IME) are neural devices that initially were designed to function as neuroscience tools to enable researchers to understand the nervous system. Over the years, technology that aids interfacing with the nervous system has allowed the ability to treat patients with a wide range of neurological injuries and diseases. Despite the substantial success that has been demonstrated using IME in neural interface applications, these implants eventually fail due to loss of quality recording signals. Recent strategies to improve interfacing with the nervous system have been inspired by methods that mimic the native tissue. This review focusses on one strategy in particular, nano-architecture, a term we introduce that encompasses the approach of roughening the surface of the implant. Various nano-architecture approaches have been hypothesized to improve the biocompatibility of IMEs, enhance the recording quality, and increase the longevity of the implant. This review will begin by introducing IME technology and discuss the challenges facing the clinical deployment of IME technology. The biological inspiration of nano-architecture approaches will be explained as well as leading fabrication methods used to create nano-architecture and their limitations. A review of the effects of nano-architecture surfaces on neural cells will be examined, depicting the various cellular responses to these modified surfaces in both *in vitro* and pre-clinical models. The proposed mechanism elucidating the ability of nano-architectures to influence cellular phenotype will be considered. Finally, the frontiers of next generation nano-architecture IMEs will be identified, with perspective given on the future impact of this interfacing approach.

## Introduction

Intracortical microelectrodes (IME) were initially designed for research purposes to enable researchers in the late 1930s an ability to improve the understanding of the nervous system (Hodgkin and Huxley, 1939; Renshaw et al., 1940; Grundfest and Campbell, 1942; Grundfest et al., 1950). The first clinical use of neural electrode technology was in 1985, when the FDA approved the use of cochlear prosthetics (Spelman, 1999). Since then, clinical implementation of IME has been employed to treat patients with numerous neurological diseases and injuries, such as amyotrophic lateral sclerosis (ALS) and spinal cord injuries (Gilja et al., 2015; Schroeder and Chestek, 2016; Ajiboye et al., 2017). Unfortunately, an impediment preventing the clinical deployment of IME technology is the complex inflammatory response occurring after electrode implantation, leading to decreased recording quality (Chestek et al., 2011; Jorfi et al., 2014; Kozai et al., 2015). The initial insertion of IME produces an injury in the local brain tissue, breaching of the blood brain barrier (BBB), eliciting an influx of chemical and biological markers, resulting in an inflammatory response (Polikov et al., 2005; Lau et al., 2013; Potter et al., 2013; Kozai et al., 2015). The early failure of IME has instigated substantial research in the development of next generation electrodes.

There are numerous types of IME, such as silicon microelectrode arrays, metal micro/nano-wires, carbon nano-tubes (CNTs), and conductive polymers, which have been categorized by their backbone material (for full reviews on these IME types, see Voge and Stegemann, 2011; Fernández and Botella, 2017). Nevertheless, biomimetic alterations and advancements have inspired the design of the most recent IME technology. Engineers and scientists reason that biomimetic alterations to create new electrodes that reflect the properties of the brain will allow for better biocompatibility of the implants *in vivo*, which may lead to improved quality and longevity of recordings (Nguyen et al., 2014; Patel et al., 2016; Chen R. et al., 2017; Ereifej et al., 2017; Wei et al., 2018). For example, Tybrandt et al. (2018) have developed stretchable electrode grids that allow for high density and high-quality chronic recordings, which reflect the modulus of the brain better than either traditional metal or silicon-based electrodes. Liu (2017) have created a flexible electrode grid, neural mesh that, once injected into the brain, unfolds and is able to record freely moving rats with stable recording quality and coherence chronically. This neural mesh is able to follow micro-movements in the brain caused by mechanical movements of the subject or growth, decreasing chances of neural shear damage, and allowing long-term recording (Liu, 2017). The latter innovation has gained significant interest, most notably, Elon Musk’s company Neuralink, has also created a similar neural mesh, called neural lace, which will allow human integration with artificial intelligence (Winkler, 2017; Wu and Rao, 2017).

Amidst the variety of biomimetic electrode types, we introduce the concept of nano-architecture in this review as a class of biomimetic surface alteration for IMEs. We use the term nano-architecture to encompass all topographical surface modifications, such as nano-grooves, nano-pillars, nano-fibers, and materials with inherent structural components. The inspiration of creating nano-architecture on IME surfaces is based on the architecture of the brain, specifically the extracellular matrix (ECM). The ECM is composed of a 3D and high-aspect ratio architecture (Wu et al., 2006; Millet et al., 2010). The 3D environment allows cells to have topographical cues which will allow them to differentiate and perform their specific functions (Kriparamanan et al., 2006). Several studies have shown that surfaces that can mimic the architecture of the natural *in vivo* environment will consequently result in an improved biocompatible response (Curtis et al., 2004; Kotov et al., 2009; Ding et al., 2010; Millet et al., 2010; Zervantonakis et al., 2011). Nano-architecture substrates indicate increase in initial protein adsorption, thus leading to subsequent attachment and proliferation of cells (Ereifej et al., 2013b; Nguyen et al., 2016). Alignment of neuronal cells in the brain have also been shown to depend on the roughness and direction of the substrate surface patterns (Khan et al., 2005; Johansson et al., 2006; Ereifej et al., 2013b; Park et al., 2016; Kim et al., 2017). Although the exact mechanism is not completely understood, it is thought that nano-architecture is able to indirectly guide the growth and alignment of neurons (Nguyen et al., 2016). Which is beneficial for IME implementation, since enabling neuron growth and proliferation near the implant may allow for improved recording quality. In addition to changes in morphology and protein adhesion, nano-architecture has also been implicated in changes to cell differentiation, phenotype, and gene expression (Kotov et al., 2009; Ereifej et al., 2013a; Yoo et al., 2015; Nguyen et al., 2016; Thompson and Sakiyama-Elbert, 2018).

The goal of the subsequent sections of this review will be to emphasis the role of the architecture with protein and cell interactions, specifically with the central nervous system cells, in order to convey the rationale behind nano-architecture approaches. The biological inspiration of nano-architecture approaches will be explained as well as leading fabrication methods used to create nano-architecture and their limitations. We will then explore the effects of nano-architecture surfaces on neural cells, depicting the various cellular responses to these modified surfaces in both *in vitro* and pre-clinical models. The proposed mechanism elucidating the ability of nano-architectures to influence cellular phenotype will be considered. Finally, the frontiers of next generation nano-architecture IMEs will be identified, with perspective given on the future impact of this interfacing approach.

## The Role of Architecture for Brain Homeostasis and Physiological Processes

In order to convey the rationale behind nano-architecture approaches, an understanding of the brain’s ECM is crucial. The brain’s ECM is made up of components created by the cells within it: neurons, astrocytes, oligodendrocytes, and microglia (Lau et al., 2013). There are three main ECM components, the basement membrane (basal lamina), the perinueonal net, and the neural interstitial matrix (Lau et al., 2013). The basement membrane, which lies around the cerebral vasculature, is composed of laminin, collagen IV, nidogen, and heparin sulfate proteoglycans (also called perlecans). These proteins support the cellular interactions between the brain capillary endothelial cells (BCECs), pericytes, and astrocytes (Thomsen et al., 2017). Collagen IV makes up about 50% of the basement membrane, and plays an essential role for the creation of suprastructures with laminin in the basement membrane (LeBleu et al., 2007). Laminin is the second most common non-collagenous protein in the basement membrane, and are vital to ensuring proper scaffolding in the ECM (LeBleu et al., 2007). Nidogen is a glycoprotein important in connecting laminins to collagen, and make up 2–3% of the basement membrane (LeBleu et al., 2007). All of the proteins in the basement membrane have been shown to demonstrate a role in the maintenance of the BBB and homeostasis (LeBleu et al., 2007; Farach-Carson et al., 2014; Thomsen et al., 2017). The perineuronal net is a lattice that wraps around neurons and brings dendrites closer to soma of neurons, creating very close synaptic contacts. It is composed of a hyaluronan backbone, chondroitin sulfate proteoglycans, tenascins, and hyaluronan and proteoglycan link proteins (Sorg et al., 2016; van’t Spijker and Kwok, 2017). Expressed late in postnatal development, the formation of the perineuronal net signals the maturation and decreased plasticity of the nervous system, while also increasing synaptic stability (McRae and Porter, 2012). Tenascins have been shown to help regulate neuron differentiation and migration, while link proteins stabilize the non-covalent binding of proteoglycans, such as chondroitin sulfate proteoglycan 1, and hyaluronan, allowing for load bearing capabilities and support (Tsai et al., 2014; Oohashi et al., 2015). Finally, the neural interstitial matrix connects neurons and vasculature, composing 15–20% of the total brain (Lei et al., 2017).

**FIGURE 1:**
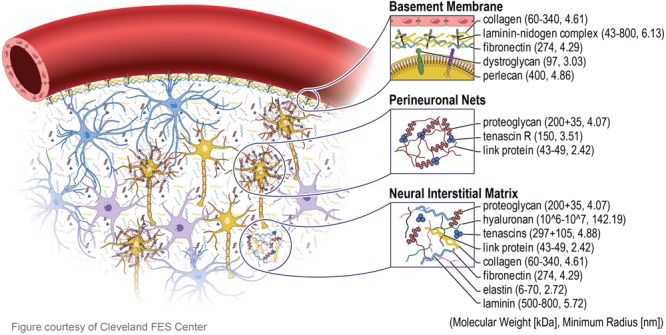
Extracellular matrix in brain. The extracellular matrix (ECM) in the brain is divided into three major components. The basement membrane (basal lamina) which lies around the cerebral vasculature, the perinueonal net which surround neuronal cell bodies and dendrites, and the neural interstitial matrix which are diffusely distributed between cells in the parenchyma. The blue, purple, and yellow cells depict astrocytes, microglia, and neurons, respectively. The proteins that make up the composition of the ECM are all tens to hundreds of nanometer in radius, inspiring the next generation nano-architecture style neural electrodes.

The three components of ECM in the brain, including the molecular weight and size of each specific protein are illustrated in **Figure 1**. It is important to note that the architecture of the individual ECM components are on the nanometer scale, a unique characteristic that is crucial to replicate onto neural implants. Nano-architecture etchings onto neural devices is thought to reduce the foreign body response by providing architectural cues for proteins and cells to respond to, that are similar to the natural ECM environment. The ECM structure and components enable cellular interactions by means of protein expression, adhesion, and cellular sensing of the ECM environment. Cells are signaled to release expression factors and non-soluble ECM molecules that are necessary for cell adhesion, proliferation, morphology, and phenotypic changes (Selvakumaran et al., 2008; Jang et al., 2010; Yoo et al., 2015; Schulte et al., 2016a; Yang et al., 2017). Likewise, a nano-architecture substrate will allow cells to sense structural cues, which triggers a similar cellular response observed when cells are in their natural environment surrounded by the ECM (Selvakumaran et al., 2008; Yoo et al., 2015; Schulte et al., 2016a; Yang et al., 2017). The nano-architecture surfaces provide cues to initiate the production of ECM molecules necessary for initial cell attachment to surfaces (Cui et al., 2018). Furthermore, the nano-architecture surface results in an increased adsorption of those ECM molecules necessary for cell attachment (Salakhutdinov et al., 2008; Ereifej et al., 2013b; Woeppel et al., 2018). These surface-cell topographical interactions initiate the cellular response that can lead to decreased inflammation around implanted IME.

The homeostasis and health of the brain relies on the proper function of all three components of the ECM. Several neurological diseases have been associated with damage to ECM proteins leading to abnormal structural and topographical deviations. Aggregations of misfolded α-synuclein (i.e., lewy bodies) are hallmarks of Parkinson’s disease (Lim and Yue, 2015; Yoo et al., 2015). Misfolded aggregates of superoxide dismutase 1 (SOD1) and transactive response DNA binding protein 43 kDa (TDP-43) are characteristic of amyotrophic lateral sclerosis (ALS) (Sahl et al., 2016; Ciryam et al., 2017). Moreover, aggregates of huntingtin (Htt) exon 1 are known hallmarks of Huntington’s disease (Sahl et al., 2016; Ciryam et al., 2017). Protein aggregation into inclusion bodies such as Lewy bodies are usually broken down via neuronal autophagy (Heras-Sandoval et al., 2014; Lim and Yue, 2015). However, the neurodegeneration observed following inflammation or trauma due to the implantation of IME may lead to a decrease in the neuronal phagosomes that enable this process to function. The architecture of misfolded proteins leads to aggregation and clumping that would normally not occur in properly folded proteins. Therefore, aberrant 3D structure of the misfolded proteins may lend insight into how proteins and other ECM components can react to a smooth surface compared to a nano-architecture surface.

Several of the hallmark protein aggregations and misfolding involved with the neural diseases described above, have also been thought to play a role in the inflammatory and oxidative stress response following neural electrode implantation. In fact, it has been shown that the implantation of neural electrodes leads to increased expression of proinflammatory and oxidative stress genes (Karumbaiah et al., 2013; Ereifej et al., 2017, 2018; Bennett et al., 2018; Falcone et al., 2018). Not only does increased oxidizing molecules such as nitric oxide (NO) from mitochondria, neurodegeneration, and the other molecules secreted in this pathway play a part in the failure of implanted electrodes, but they may also play a part in the generation of inclusion bodies leading to neuronal death. Thus, it is imperative to control the inflammatory and oxidative stress response, as well as the protein adhesion and conformation around implanted neural electrodes. Perhaps, electrodes with nano-architecture inspired by the healthy ECM neural tissue may play a role in mitigating the inflammatory and oxidative stress response. The hypothesis is that implants with architectures similar to the architectures of the native brain environment, will provide cells an opportunity to maintain their quintessential inactivated phenotypes after electrode implantation. Chronic foreign body response can be controlled, health of neurons can be maintained and high-quality chronic recordings can be enabled. There are limited technologies that can incorporate nano-architecture onto neural electrodes due to the desired size of surface features, the geometry, material, and manufacturing needs of the electrode.

## Technologies to Create Nano-Architecture

The addition of nano-architecture onto neural implant surfaces has the ability to improve the biocompatibility as well as induce varying morphology and phenotype of the cells around them. Therefore, fabrication methods that yielded high accuracy and reproducibility to create nano-architectures are imperative. Some fabrication techniques to incorporate nano-architecture on device surfaces include, focused ion beam (FIB) etching to create nano-grooves, electron beam lithography (EBL) to create nano-lines, and a combination of photolithography and nano-sphere lithography to make nano-structures (Ereifej et al., 2017; Kim et al., 2017; Nissan et al., 2017). Furthermore, there are nano-scale materials that can also be utilized to create nano-architecture, such as CNTs, nano-wires, and bio-inspired materials (Christopherson et al., 2009; Dugan et al., 2010; Fernández and Botella, 2017). The following sections will discuss some of the commonly used fabrication techniques and materials utilized to create nano-architecture.

### Fabrication Methods to Create Controlled Nano-Architecture

Nano-architectural effects on implant interface have been investigated for silicon, titanium, and some polymethyl methacrylate (PMMA)-based electrodes. Many methods can be used to create the nano-architecture on substrates, such as photolithography, optical lithography, soft lithography, nano-sphere lithography, and nano-stencil (Blattler et al., 2006; Kriparamanan et al., 2006; Das et al., 2008; Ding et al., 2010). The benefits of using fabrication methods discussed below, is to create specific patterns, geometries, and sizes that are reproducible. **Table 1** summarizes the characteristics of the fabrication methods discussed below.

**Table 1:** Summary of different nano-architecture fabrication techniques.

Nano-architecture technique	Compatible materials	Resolution	time required	Cost	Serial or batch processing	Features	References
Electron beam lithography	Silicon and conductive materials. Requires electron-sensitive resist (i.e., PMMA)	Below 10 nm	Slow for scanning focused electron beam	High-equipment cost (>$1 million)	Serial for focused electron beam; batch processing possible for projection electron beam lithography; device-scale or wafer-scale	Often used to create master mold for nano-imprint lithography	Tseng et al., 2003; Dong et al., 2005a; Joo et al., 2006; Lee et al., 2007; Dalby et al., 2008; Yang and Leong, 2010; Scholten and Meng, 2016
Nano-imprint lithography	Silicon-based materials, metals, polymers	2–100 nm	Relatively fast to transfer pattern from mold to resist	High cost of master mold, but overall cost is relatively low due to reusability of mold	Batch processing; device-scale or wafer-scale	Two broad categories: thermal NIL and ultraviolet NIL	Chen and Ahmed, 1993; Schulz et al., 2000; Dong et al., 2005b; Guo, 2007; Li et al., 2011; Al-Abaddi et al., 2012; Choi et al., 2013; Eom et al., 2015; Baquedano et al., 2017
Focused ion beam lithography	Silicon-based materials, metals, and polymers	∼20 nm	Slow rate of milling	High-equipment cost (>$1 million)	Serial processing on device-scale	Direct write; flexible design and materials	Watkins et al., 1986; Veerman et al., 1998; Lehrer et al., 2001; Reyntjens and Puers, 2001; Heyderman et al., 2003; Gabay et al., 2005; Kim et al., 2007; Lanyon and Arrigan, 2007; Raffa et al., 2008; Christopherson et al., 2009; Ziberi et al., 2009; Bechara et al., 2010; Dugan et al., 2010; Menard and Ramsey, 2010; Wang et al., 2017; Vermeij et al., 2018

#### Electron Beam Lithography

**FIGURE 2:**
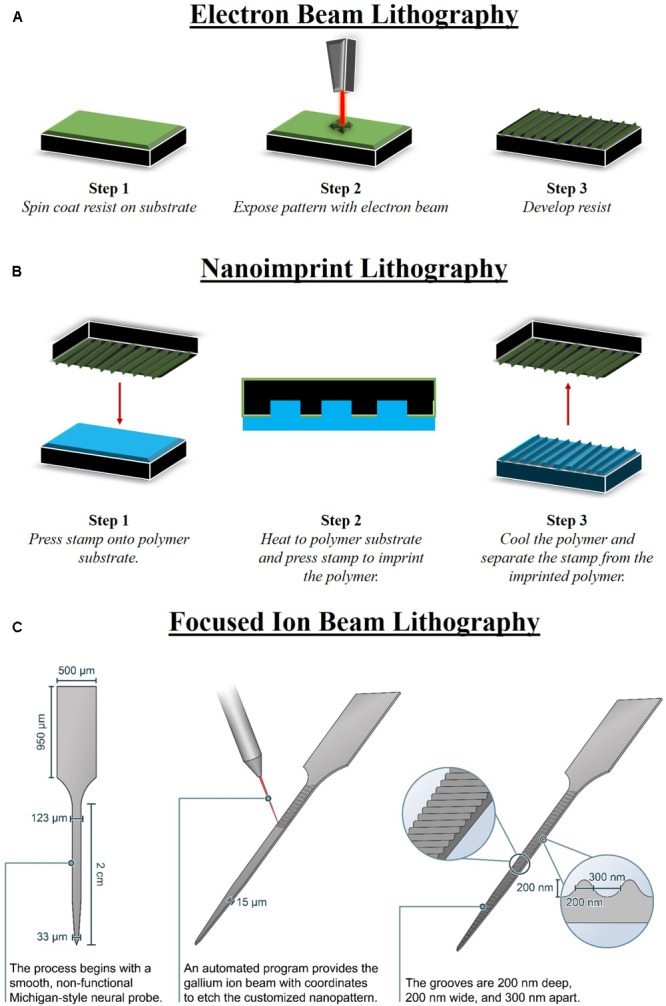
Methods to fabricate nano-architecture. There are limited methods that are able to fabricate nano-architecture features in specific patterns, geometries, and sizes that are reproducible. **(A)** Electron beam lithography (EBL) consists of three major steps, including spin coating a resist on the substrate, electron beam to expose the pattern, and finally develop the resist. A prominent use for EBL is fabricating master mold that can be used with other fabrication processes, such as nano-imprint lithography (NIL). **(B)** NIL can be performed on a larger area than EBL and also involves three major steps consisting of pressing the EBL stamp into the polymer, heating the polymer to imprint the stamp, and then finally cooling and releasing the stamp from the imprinted polymer. **(C)** An advantage of focused ion beam lithography (FIB) is that it can produce nano-architecture features on devices of various shapes. FIB can be used to fabricate nano-architecture on neural devices post-processing, directly onto the manufactured device (Ereifej et al., 2017).

Electron beam lithography is a nano-fabrication technique in which an electron-sensitive resist is selectively exposed to an electrode beam to form a high-resolution pattern. EBL can achieve resolutions less than 10 nm when used in a maskless scanning configuration that uses a highly focused electron beam (Tseng et al., 2003; Dalby et al., 2008; Yang and Leong, 2010). Alternatively, throughput can be increased at the expense of resolution when a diffuse electron source is projected through a thin mask to expose the resist (Tseng et al., 2003). After spin-coating the resist to a thickness of 50–500 nm on the substrate surface and exposing to the electron beam, the resist is developed in a solvent to remove unwanted material (**Figure 2A**). The nano-architectural pattern can then be transferred to the underlying substrate using standard etching techniques. PMMA is the most commonly used positive resist for EBL, though negative EBL resists are also used (Tseng et al., 2003). Positive resists become more soluble after electron beam exposure, while negative resists form crosslinks after electron beam exposure.

Electron beam lithography is most compatible with patterning silicon or thin metal films that can dissipate the excess charge from the electrode beam (Lee et al., 2007; Dalby et al., 2008; Scholten and Meng, 2016). Insulating films are more challenging to pattern with EBL because electrons that pass through the resist become trapped at the substrate surface, forming variations in resist surface potential. The electrons can then be deflected and result in distorted patterns (Joo et al., 2006). However, researchers have developed methods to enable EBL on insulating glass substrates by controlling the beam energy and on polymer substrates by exposing through a thin conductive metal film (Joo et al., 2006; Scholten and Meng, 2016).

Electron beam lithography requires high-cost instrumentation, and throughput is limited for high-density features due to the serial nature of the exposure and low sensitivity of the resist to the electron beam (Yang and Leong, 2010). An advantage of EBL is its ability to be used to fabricate a single master mold that can be reused several times [e.g., for nano-imprint lithography (NIL)] (Dong et al., 2005a).

#### Nano-Imprint Lithography

Nano-imprint lithography relies on the contrast in a resist thickness produced by mechanical deformation when pressed with a rigid mold or stamp with a pre-defined relief structure. The mold is typically fabricated by patterning Si or SiO_2_ by combining other nano-fabrication tools and techniques, such as EBL, with reactive ion etching techniques. The resist layer is spin-coated onto the sample surface. In contrast to EBL, the mechanical properties of the resist layer are of prime importance as the Young’s modulus of the resist must be lower than the Young’s modulus of the mold. In hot embossing NIL, thermoplastic resists are imprinted with the mold at temperatures 70–90° above *T_g_*, then cooled before releasing from the mold (**Figure 2B**) (Guo, 2007; Choi et al., 2013). Alternatively, low-viscosity UV-curable resists can be cured by UV light at ambient temperatures while being imprinted with the mold (Schulz et al., 2000; Guo, 2007). After forming the relief pattern in the resist, the underlying substrate can then be etched using standard processes (Al-Abaddi et al., 2012). By offering a variety of resist materials with a range of fabrication conditions, NIL can be used to pattern nano-architectures on a variety of substrate materials, including silicon and polymers (Eom et al., 2015; Baquedano et al., 2017). Resolutions less than 100 nm and even approaching 2 nm have been achieved using NIL (Dong et al., 2005b; Li et al., 2011).

Nano-imprint lithography can be performed on a larger area than EBL or FIB, providing an efficient and cost-effective method of nano-scale patterning at the wafer scale (Chen and Ahmed, 1993; Al-Abaddi et al., 2012). Additionally, the same mold can be used to pattern multiple samples, and therefore is more cost-effective than serial nano-patterning methods (Al-Abaddi et al., 2012).

#### Focused Ion Beam Lithography

Focused ion beam nano-machining technology is a direct–write technique for selectively ablating the substrate surface with a finely focused, high-current ion beam (Lehrer et al., 2001; Raffa et al., 2008). FIB can produce a wide variety of features with nano-scale resolution and high-aspect ratio (Watkins et al., 1986; Veerman et al., 1998; Raffa et al., 2008). The magnitude of the ion beam current modulates the ion beam spot size within a range of 3 nm to 2 μm (Lehrer et al., 2001; Raffa et al., 2008). A primary advantage of FIB is that it can be used on a wide variety of materials, including silicon, metals, and polymers (Veerman et al., 1998; Lanyon and Arrigan, 2007; Ziberi et al., 2009; Menard and Ramsey, 2010). Additionally, FIB can be applied on non-planar surfaces and can be used for post-processing on individual devices (**Figure 2C**) (Reyntjens and Puers, 2001). Users must be cautious, however, as ion implantation and re-deposition of ablated material can lead to damaged nano-structures or structures that do not match the intended geometry (Vermeij et al., 2018). Overall, FIB allows for a high degree of control and flexibility in feature geometries, compatible materials, and surface requirements, at the expense of a slow throughput rate that limits the potential for mass production (Heyderman et al., 2003).

### Nano-Scale Materials Producing Nano-Architecture

In addition to the pre-defined nano-architecture fabrication techniques described above, nano-scale topography can also be applied to biomedical devices by depositing or growing nano-scale materials on a substrate. At the nano-scale, exact geometries cannot be defined to the extent possible using EBL or FIB due to randomness in orientation, size, and positioning. However, at the device scale, feature size, and density can be controlled. Several reviews discuss these materials in great depth, and therefore we will only briefly summarize some of the nano-scale materials (Kotov et al., 2009; Shah, 2016; Wang et al., 2017). CNTs are hollow carbon tubes with a nanometer-scale diameter and appealing electrical, mechanical, and biological characteristics (Wang et al., 2017). Gabay et al. (2005) used iron-based nanoparticles as catalysts for CNT growth by chemical vapor deposition. Neurons demonstrated a strong affinity to grow on the CNT clusters and send out neurites to connect clusters (Gabay et al., 2005). Polycaprolactone was extruded through aluminum oxide membranes to form high-aspect ratio nano-wires for neural tissue engineering applications (Bechara et al., 2010). Silicon nano-wires can be produced by chemical vapor deposition epitaxial growth or by etching, and produce very high-aspect ratio structures oriented perpendicular to the substrate (Kim et al., 2007). Electrospinning can be used to cover a substrate with polymer nano-fibers 200–1500 nm in diameter (Christopherson et al., 2009). Bio-related materials, such as cellulose nano-whiskers of 10–15 nm diameter, have formed nano-topographies by spin-casting onto a substrate (Dugan et al., 2010). Some of the nano-scale materials also offer unique advantages to improve the functional qualities of implanted neural devices, such as reducing neural recording electrode impedance. The nano-scale materials can be used to cover a large area without necessitating specialized serial patterning equipment, thus lending these strategies to larger-scale manufacturing.

## Nano-Architecture Effect on Neural Cells (*In Vitro*)

Electrode implant performance and success depends heavily on the reaction of the cells within the local and surrounding areas of the brain. While the initial inflammatory response is beneficial for maintaining homeostasis, chronic inflammation may lead to glial scarring, neurodegeneration, and oxidative stress. All of these events may lead to failure of the implant via mechanical breakdown of the implant or reduced to no signal recording (Polikov et al., 2005; Kang et al., 2011; Kozai et al., 2015; Potter-Baker et al., 2015; Bennett et al., 2018; Ereifej et al., 2018). It has been shown that protein adsorption onto the surface of the implant plays a large role in the behavior and response of the cells (Ereifej et al., 2013b; Nguyen et al., 2016). To reiterate, although the exact mechanisms are unknown, cells are able to respond to various architecture geometries within their environment, leading to changes in differentiation, morphology, phenotype, gene expression, signaling molecules, cytokines, and protein production (Frimat et al., 2015; Marcus et al., 2017; Skoog et al., 2017; Eyster and Ma, 2018b). Tanaka and Maeda (1996) showed that substrate surface-cell topographical interactions may influence neural cell response more than the surface chemistry interactions. When microglia cells were seeded on living astrocyte monolayers, fixed astrocyte monolayers, and a glass coverslip, 80% of microglia showed ramification and processes elongation on the fixed and living astrocyte monolayers while almost no microglia showed ramification on the coverslip (Tanaka and Maeda, 1996). Similarly, Chen et al. (2010) showed that 500 nm parallel grooves imprinted onto poly(s-caprolatctone), poly(lactic acid), and poly(dimethylsiloxane) was able to reduce the foreign body response in macrophage behavior, independent of the material’s chemistry. The implications of these studies highlight the importance of nano-architecture to enhance positive cell-implant interactions. The ECM of the brain inspires nano-architecture approaches, aiming optimize the protein adsorption, and the subsequent response of the cells. Therefore, it is important to explore the effect of nano-architecture on the primary types of cells present in the brain: neurons, astrocytes, and microglia. It is important to note that nano-architecture substrates will elicit varying cellular responses depending on the cell type. This is an important finding for neural electrode implementation, because the goal of optimal neural electrodes is to improve the neural attachment without negatively affecting glial cell activation. The following subsections will discuss the effects of nano-architecture on neural cells.

### Role of Nano-Architecture on Astrocytes

Astrocytes are the most abundant glial cell in the brain, bridging neurons to the vasculature of the CNS. Astrocytes maintain the BBB and homeostasis of the brain via secreted factors that either promote or disrupt barrier development (Herndon et al., 2017). During inflammation, astrocytes regulate the breakdown of the basement membrane to allow infiltrating macrophages and other immune cells access to the effected site (Herndon et al., 2017). In addition to the regulatory and health-sustaining role of astrocytes, it has been shown that neurons grow preferentially according to the track provided by astrocytes, effectively guiding the growth, and alignment of neurons (Sofroniew, 2009; Hsiao et al., 2015). Since the quality of the recorded signal is dependent on the distance of the neuron to the electrode, it may be beneficial to have astrocyte adhesion onto an implant surface, so that neurons will align in favorable positions.

Initial protein adsorption onto the implant surface has been implicated in the adhesion and proliferation patterns of astrocytes. Ereifej et al. (2013b) showed that reduced protein adsorption resulted in a reduction in glial fibrillary acidic protein gene expression from astrocytes cultured onto PMMA nano-grooved surfaces 200 nm deep and either 277 nm wide or 555 nm wide compared to non-patterned surfaces. Additionally, Ereifej et al. (2013b) found an increase in fibronectin and collagen adsorption rate onto the nano-patterned surfaces, suggesting a change of protein conformation, due to the increase of astrocyte adhesion onto the nano-patterned substrates. As protein adsorption is an important initial factor for astrocyte adhesion and proliferation, groups have begun studying the effects of coating proteins onto implant surfaces and studying those effects on astrocytes. Commonly used proteins used in the effort to increase favorable astrocyte-implant interactions are those found naturally in the brain ECM, such as fibronectin and collagen. These have been used to coat and create nano-architecture for increasing astrocytic adhesion and inactivation. Zuidema et al. (2014) used electrospun polylactic acid (PLLA) fibers randomly oriented (2.38 ± 0.46 μm average diameter) and aligned (2.49 ± 0.32 μm average diameter), coated with fibronectin as a substrate to seed astrocytes in order to direct astrocyte migration and extension. This study exhibited the aligned fibers directed astrocytic migration, which is thought to positively modify the neuroprotective properties of glial cells (Zuidema et al., 2014). Chen P. et al. (2017) summarized that astrocytes tend to align in the direction of the aligned fibers and have a rectangular morphology, compared to the circular morphology of astrocytes seeded on randomly oriented fibers. Moreover, Frimat et al. (2015) showed that nano-grooves 108 nm high cut into polydimethylsiloxane (PDMS) are likewise an ideal approach to align and guide astrocytes as they migrate.

**FIGURE 3:**
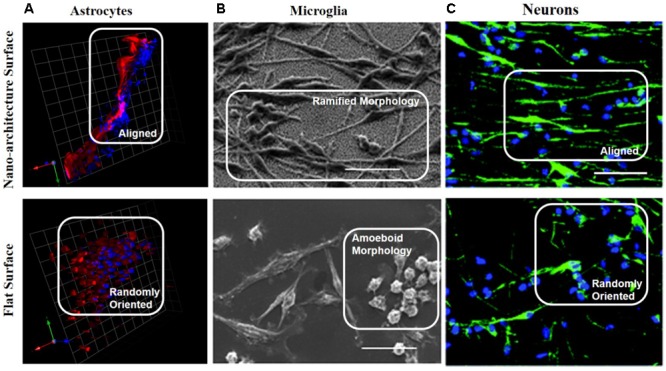
Neural cells cultured with nano-architecture substrates alter cellular morphology and phenotype. **(A)** Nano-patterned PDMS pins cultured in organotypic brain slices revealed astrocytes (red) and nuclei (blue) aligned along the nano-patterned direction, but were randomly oriented when cultured with the non-patterned PDMS pins. This change in cellular morphology resulted in reduced levels of inflammatory gene expression GFAP, IL-1β, TGFβ1, and TNFα, from organotypic brain slices cultures with nano-patterned pins compared to non-patterned pins (Ereifej et al., 2013a). Scale bar is 25 microns. Reprinted from Ereifej et al. (2013a). **(B)** Scanning electron images of microglia cultured on top of nano-architecture (top) or smooth (bottom) silicon substrates. The microglia seeded on nano-architecture substrates presented more ramified morphology compared to the amoeboid morphology shown on the flat substrates. Microglia on nano-patterned substrates exhibited decreased activation phenotypes and increased adhesion (Persheyev et al., 2011). Scale bar is 5 microns. **(C)** Neuronal stem cells (NSC, green stained for neurons, blue stained for cell nuclei) cultured on titanium coated nano-architecture substrates displayed aligned morphology along the nano-grooves. NSC on the nano-architectures exhibited higher ratio of differentiation into dopaminergic and glutamatergic neurons compared to NSC cultured on flat substrates. Scale bar is 50 microns (Yang et al., 2017). Reproduced in part from Yang et al. (2017) with permission of The Royal Society of Chemistry. License number 4332650271766.

An implication of astrocytes cultured on nano-architecture substrates altering their cellular morphology and adhesion is that they may also exhibit altered phenotype to help guide subsequent neuron growth and migration closer to the nano-architecture implant. Ereifej et al. (2013a) revealed astrocytes near nano-patterned PDMS substrates (150 ± 2 nm depth, 117 ± 11 nm ridge length, and 170 ± 16 nm groove width) had decreased neuroinflammatory markers and cytokines. Astrocytes in organotypic brain slices, cultured with nano-patterned substrates, aligned along the nano-pattern grooves, thus altering the cell morphology and downstream phenotype (**Figure 3A**). The astrocytes cultured with nano-patterned substrates exhibited decreased expression GFAP, TNFα, and IL-1β, which are important factors in chronic inflammation (Ereifej et al., 2013a). The mechanism behind this phenomenon is discussed in Section “Proposed Mechanism of Cellular Response to Nano-Architecture Surfaces” of this review. As can be seen collectively from these studies, nano-architecture surfaces can be pivotal in reducing the pro-inflammatory cytokines and activated astrocytes occurring after neural electrode implantation.

### Role of Nano-Architecture on Microglia/Macrophages

At the onset of IME insertion, resident microglia activate and the compromised BBB allows for infiltrating macrophages to enter the local damaged tissue (Ravikumar et al., 2014). Microglia and macrophages form the first line of defense against invading pathogens via phagocytosis and release of cytotoxic molecules, cytokines, reactive oxygen intermediates, proteinases, and complement proteins (Thameem Dheen et al., 2007; Li and Barres, 2017). Chronic activation of microglia and macrophages leads to neurodegeneration (Thameem Dheen et al., 2007; Li and Barres, 2017). In response to the detrimental outcomes of chronically activated microglia and macrophages around implanted IME, a consensus desire has grown to reduce the level of activated microglia and macrophages from binding to substrate surfaces. In this venture, nano-architecture has been explored as a way to control activation of microglia and macrophages and decrease inflammatory molecule release.

For example, Luu et al. (2015) found that macrophages cultured on nano-groove titanium surfaces with widths ranging from 400 nm to 5 μm had an elongated morphology aligned with the nano-pattern and trended toward an anti-inflammatory phenotype depicted by significantly higher expression of interleukin 10 (IL-10), and anti-inflammatory cytokine. Notably, Persheyev et al. (2011) observed elongation of BV-2 microglia cells and increased actin-rich microdomains when cultured on substrates with nano-spikes <70 nm, indicating a ramified quintessential phenotype (**Figure 3B**). Additionally, Saino et al. (2011) found that the secretion of proinflammatory molecules from microglia was dependent on the diameter of the PLLA fibers they were cultured on. In fact, PLLA scaffold with nano-fibers (diameters 0.61 ± 0.18 and 0.55 ± 0.16 μm for random and aligned fibers, respectively) reduced the level of proinflammatory molecules compared to the same scaffold with microfibers (diameters 1.53 ± 0.32 and 1.60 ± 0.25 μm for random and aligned fibers, respectively) or a flat PLLA film (Saino et al., 2011). Similarly, Pires et al. (2015) discovered that microglia cultured on electrospun poly(trimethylene carbonate-co-𝜀-caprolactone) fibers with 1.09 ± 0.1 μm diameter exhibited elongated morphology signifying decreased activation, compared to a flat surface of the same chemistry. They additionally showed that when media from the microglia culture was introduced to astrocytes, astrogliosis was not exacerbated, further signifying the microglia were not activated (Pires et al., 2015). Cumulatively, these studies indicate the promise of nano-architecture to influence microglia and macrophage phenotype. This observation can be translated to IME implanted into the brain, thus potentially reducing the glial cell activation, chronic inflammation, and neurodegeneration.

### Role of Nano-Architecture on Neurons

Neuron distance from the electrode determines the quality of the recorded signal (Buzsáki, 2004). Chronic inflammation may lead to decreased neuronal density around the implant due to neuronal death (Jorfi et al., 2015). Therefore, it is crucial to guide neurons and keep neuronal density around the implant high. As discussed earlier, initial protein adsorption causes astrocyte adhesion, which neurons grow over according to the tracks laid by astrocytes. Neuron growth cones are able to sense the architecture in the environment due to mechanotransductive components in the cell membrane, which signal to the cytoskeleton (Section Proposed Mechanism of Cellular Response to Nano-Architecture Surfaces explains this mechanism in detail). This mechanotransductive pathway leads to specific growth patterns exhibited when neurons are cultured onto nano-architecture substrates (Nissan et al., 2017; Eyster and Ma, 2018b).

Growth cones are called so because they resemble cones on the tips of extending neurites. Jang et al. (2010) found that when neurons were seeded on a surface with nano-grooves 350 nm wide and 350 nm high, fillopodia at the growth cone tips aligned along the direction of the patterns. Frimat et al. (2015) have explored the response of neurons seeded on a substrate with a nano-patterned surface made of PDMS nano-grooves 108 nm high. They found neurite extensions aligning to the patterns, as well as alignment of the soma of neurons (Frimat et al., 2015). Additionally, Xie et al. (2016) found that 47% of neurons seeded on a nano-patterned microseive array with nano-grooves 230 nm wide with a period of 600 nm showed alignment along the direction of the grooves. Recently, Woeppel et al. (2018) found that roughening the silicon substrate surface with silica nanoparticles 60 nm in diameter on average, followed by L1 protein adhesion lead to increase neuron outgrowth. Notably, Nissan et al. observed an increase in the number of neurites and branching points toward more complicated structures, when neurons were seeded onto silver nano-lines (180–500 nm wide, 160 nm high, and 700 nm apart) made via EBL. However, they found that the neurons consistently aligned at a ∼45° angle to the nano-lines (Nissan et al., 2017). This is thought to be due to how neurons form focal adhesions on the different surfaces. Neural fillopodia extend from the neural cell body and probe around its environment with focal adhesions to see if the environment is suitable for adhesion and growth (Marcus et al., 2017; Eyster and Ma, 2018b). Focal adhesions signal to intracellular cytoskeletal components such as talin and paxillin as a result of surface–protein interactions, which lead to directional growth (Eyster and Ma, 2018b). Due to the relationship between focal adhesions and cytoskeletal components, the organizational structure of focal adhesions may be the reason for neuron cell alignment (Eyster and Ma, 2018b).

Nano-architecture has also been implicated with cell differentiation via changes in gene expression. Yoo et al. (2015) found that seeding fibroblasts on substrates with nano-grooves 300 nm wide and 400 nm apart led to cell alignment and expression of dopamine markers, leading to reprogramming of fibroblasts into functional dopaminergic neurons. The dopamine expression led to gradual acquisition of dopaminergic neuronal characteristics, and then full differentiation into this class of neuron (Yoo et al., 2015). Additionally, Yang et al. (2017) found that seeding neural stem cells onto a conductive nano-groove substrate with groove sizes from 150 to 300 nm, led to alignment and neural guidance via increased focal adhesions and cytoskeletal rearrangements (**Figure 3C**). The neural stem cells then exhibited enhanced differentiation and maturation as elevated levels of neurite extension and neural markers Tuj1 and NeuN were observed (Yang et al., 2017). In comparison, those neural stem cells seeded on a non-patterned or non-conductive coated substrate showed lower levels of neurite extension and neural markers (Yang et al., 2017).

Collectively, the implications of nano-architecture on astrocyte, microglia/macrophage and neuron cells’ morphology, phenotype and differentiation may lead to advents of next generation IME design. The studies reviewed in this section suggest nano-architecture has the ability to control glial cell activation by reducing the expression of inflammatory markers and maintaining cellular morphology to a ramified quintessential phenotype. Remarkably, nano-architechture has been shown to increase neuron adhesion and extension, which may translate to increased neuron density around implanted IME. The combined results from the aforementioned studies advocate the use of nano-architecture to control the cellular response to biomaterials, however, conclusions specifying the exact nano-architecture to elicit a particular cellular response cannot currently be determined. Given that each study utilized a distinctive biomaterial, differing from other studies examining similar geometries of nano-architecture, it is problematic to establish an optimal nano-architecture, unbiased of the biomaterial, for a precise cell function. Thus, the imperativeness for performing studies evaluating the various nano-architectures described within this review, on the same biomaterial, would greatly benefit the field. Identification of nano-architectures to control cellular response is necessary to inform the proper design of next generation IME. The translation of the aforementioned *in vitro* studies to pre-clinical models has been nominal, but the few studies achieved proved insight for future IME designs.

## Nano-Architecture Effect on Neural Cells (*In Vivo*)

Originally utilized with tissue engineering applications, nano-architecture approaches have been employed with orthopedic and organ replacement technologies to serve as scaffolds that promote cell adhesion and viability (Karazisis et al., 2016, 2017). The potential of nano-architecture to improve biocompatibility and integrate IME in the neural tissue has become increasingly apparent. One of the proposed goals of applying nano-architecture onto the surfaces of IME has been to mitigate neuro-inflammation. Unfortunately, there has not been substantial translation of the *in vitro* findings [see Nano-Architecture Effect on Neural Cells (*In Vitro*)] to pre-clinical models. There is a gap in the literature evaluating the effects nano-architecture with IME on the inflammatory response and recording quality *in vivo*. **Table 2** highlights selected findings of next generation neural probes utilizing nano-architecture features implanted pre-clinically.

**Table 2:** Highlights of selected studies implanting nano-architecture neural probes.

Nano-architechture method	Material	Nano-architechture location	Nano-architechture specifications	Outcomes	Reference
Ion-beam assisted deposition (IBAD) anodic stain etching	Porous silicon thin-film around a ceramic electrode	Whole electrode	1 μm–100 nm sized pores (non-uniform distribution)	Enhanced neurite outgrowth while at the same time decreased astrocyte adhesion	Moxon et al., 2004
Focused gallium ion beam	Silicon	Whole electrode	Parallel grooves 200 nm wide spaced 200 nm apart, 200 nm deep	Increase neuron density 150 μm from the electrode and decreased gene expression of proinflammatory and oxidative stress associated genes at 4 weeks	Ereifej et al., 2017
Low-pressure chemical vapour deposition (LPCVD) and photolithography	Black poly-silicon and silicon	Whole electrode	520–800 nm long nano-pillars, diameter of 150–200 nm	Increased neuronal viability near the electrode at 8 weeks	Bérces et al., 2016
Anodization	Indium titanium oxide (ITO)	Whole electrode	Nanoparticles mean diameter 89 nm (random distribution)	Significant decrease of microglia and reactive astrocytes and increase of neurons. Downregulations in cleaved spectrin, a key astrocytic activation protein	Vallejo-Giraldo et al., 2017
Electrospining pedot	Pedot	Contacts	500 nm diameter fibers randomly oriented	Decreased impedance and increased SNR after 7 weeks	Abidian et al., 2007

### Role of Nano-Architecture Mitigating Neuroinflammation *in Vivo*

**FIGURE 4:**
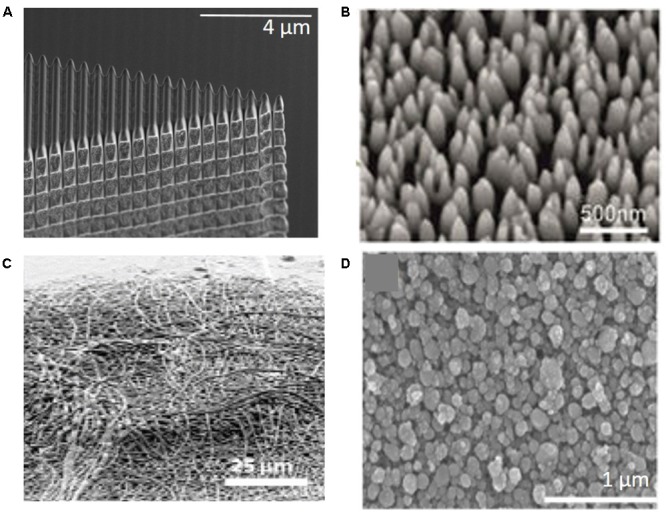
Next generation neural electrodes utilizing nano-architecture. **(A)** Focused gallium ion beam etched parallel grooves onto silicon neural probe lead to increased neuron density around implant and reduced inflammatory and oxidative stress gene expression at 4 weeks post-implantation (Ereifej et al., 2017). **(B)** LPCVD nano-pillar silicon electrode showed increased neuronal density, but did not have effect on glial cell activation (Bérces et al., 2016). **(C)** Microelectrodes with electrospun randomly oriented PEDOT, coated on the contacts, showed significantly lower impedance and higher SNR 7 weeks post-implantation (Abidian et al., 2007). **(D)** Nano-structures anodized into indium tin oxide (ITO) electrode surface revealed increased neural cell survival, modulated formation of glial scar, and promoted neural network activity (Vallejo-Giraldo et al., 2017).

He et al. (2006) demonstrated that nano-structured laminin coatings can limit astrocytic encapsulation of implanted neural electrodes at both 1 day and 4 weeks post-implantation in mice. Although these nano-structured probes showed increased microglia/macrophage activation at 1 day post-implantation, significantly decreased activation was seen when compared to the smooth silicon controls at 4 weeks. Another approach was investigated by Ereifej et al. (2017) involving a gallium ion beam etched nano-pattern (200 nm wide, 200 nm deep, and 300 nm apart) on silicon electrodes that significantly increased neuronal viability 100–150 μm from the implant site at 4 weeks post-implantation (**Figure 4A**). Additionally, tissue implanted with the nano-patterned probes had lower expression of pro-inflammatory genes at 4 weeks compared to the smooth control group (Ereifej et al., 2017). Bérces et al. (2016) demonstrated similar outcomes, observing no chronic differences for astrocytic and microglial activation, but an increase in neuronal viability around silicon electrodes nano-patterned through low-pressure chemical vapor deposition. These electrodes exhibited a nano-pillar structure 520–800 nm long with 150–200 nm diameters along the entire surface of the electrode that they found to be more biocompatible than a similar structure on the microscale (**Figure 4B**). Notably, Moxon et al. (2004) demonstrated a 70–90% nano-porous silicon-based electrode can confer reduced gliosis and increased neuronal viability utilizing an anodic etching method which provides a porous silicon thin film over ceramic electrodes. Testing over chronic recording time points showed the nano-structured coating created no deviation from proper functioning of the electrode nor altering of electrical properties of recording sites. Collectively, these pre-clinical studies indicate that the application of nano-architecture onto the IME surface can reduce glial cell activation and increase neuron density, suggesting potential improvement of recording quality.

### Role of Nano-Architecture for Improving Recording Quality

Nano-architecture on neural electrode surfaces can confer many potential electrophysiological benefits including improved recording quality, greater stimulation efficiency, and the ability to use smaller, less invasive electrodes (Heim et al., 2012). It has been shown inflammation can limit the quality of recordings from neural electrodes due to astrocytic encapsulation and microglial associated oxidative stress that limits the detection of single neurons (Nolta et al., 2015). To combat this inflammatory response, research is aimed at minimizing electrode geometry. Vitale et al. (2015) showed how CNT yarn electrodes utilize their unique microscale properties to improve biocompatibility which allows for microscale recording and stimulating electrode arrays with small contact surface areas with lower impedance than metal electrodes of similar size. They showed 15 times lower impedance resulted from CNT electrode compared to platinum iridium control electrodes. Further, despite not improving signal to noise ratio (SNR), the CNT structure revealed increased biocompatibility, which enabled increased recording quality and longevity. Although minimizing the IME geometry may seem promising, there are challenges that correspond to the decreased contact surface area.

Smaller contact surface area, results in higher noise and increased impedance, as well as lower SNR and recording quality. Therefore, increasing contact site surface area by nano-architecture modifications may offer an elegant solution to improve recording signal quality. Examples of nano-architecture modifications include, porous platinum black, golden nano-flakes or -pillars, CNTs, conducting polymers such as polypyrrole and poly(3,4-ethylenedioxythiophene):poly (styrenesulfonate) (PEDOT:PSS) (Venkatraman et al., 2011; Furukawa et al., 2013; Sessolo et al., 2013; Kim et al., 2014; Pas et al., 2018). Abidian et al. (2007) investigated PEDOT coated contacts on electrodes implanted into rats that demonstrated significantly lower impedance and higher SNR 7 weeks post-implantation (**Figure 4C**) (Abidian et al., 2007). These benefits of nano-patterned contacts showed by Abidian et al. (2007) met expectations and opened the door for the investigation of other methods to utilize nano-scale alterations and fabrication methods to improve recording contacts. Brüggemann et al. (2011) investigated a gold nano-pillar sturcture (300–400 nm high, 60 nm diameter) to increase contact surface area which lowered impedance and improved extracellular recording performance *in vitro*. Vallejo-Giraldo et al. (2017) showed that alteration of the nano-pattern on electrodes using anodized indium tin oxide (ITO) can be modulated to find different levels of glial activity, neural cell survival, and promotion of neural network activity *in vitro*. They concluded that anodized ITO electrodes with nano-structure features can be employed to deposit insulator and charge carrier regions within the same electrode system (**Figure 4D**) (Vallejo-Giraldo et al., 2017). Piret et al. (2015) found that by using a CNT scaffold to grow nano-structured boron-doped diamond coatings on neural electrode contacts, the impedance was lowered and the recording quality *in vitro* was improved. This structure included another nano-pillar structure similar to other discussed above with 500 nm a diameter of and one micron height (Piret et al., 2015). Cummulatively, the aforementioned studies indicate that the application of nano-architecture on electrode contacts shows promise to improve recording quality of neural electrodes.

Overall, these *in vivo* studies suggest a role for nano-architecture to reduce neuroinflammation surrounding implanted neural electrodes and improve rescoring quality. It would be beneficial to see future studies comparing various size and geometries of these nano-architectures to provide insight into the optimal surface for reducing inflammation. Furthermore, it would be remarkable to examine and compare the various fabrication methods to create the desired nano-architectures onto neural devices to identify best practices for manufacturing electrodes with nano-architected surfaces.

## Proposed Mechanism of Cellular Response to Nano-Architecture Surfaces

Although the exact mechanisms are not well understood, nano-architecture is thought to affect the cellular response directly or indirectly via the effects of protein adsorption onto the implant surface (Kriparamanan et al., 2006). It has been shown that nano-architecture effects protein adsorption, with various surface geometries and sizes having different rates, amounts, and conformation of adsorbed protein (Kriparamanan et al., 2006). For example, surfaces with 4 nm height, had low-randomly oriented protein adsorption, but surfaces with a height ranging from 1 to 2 nm, had very high-protein adsorption (Kriparamanan et al., 2006). Additionally, cell morphology is effected by the nano-architecture of the implant surface, as the actin filaments and focal adhesion structures generally align along the direction of the grooves, depending on the cell type (Schulte et al., 2016a). Following cell seeding onto nano-architecture surfaces, actin aggregation was observed, followed by microtubule alignment (Selvakumaran et al., 2008; Yoo et al., 2015; Yang et al., 2017). This showed that one of the first events to occur after seeding is the rearrangement of the cytoskeleton (Selvakumaran et al., 2008; Yoo et al., 2015; Yang et al., 2017). Teixeira et al. (2003) showed that surfaces with 70 nm wide grooves seeded with human corneal epithelial cells aligned themselves according to the direction of the grooves, while the same type of cells seeded in a non-textured surface did not show alignment in any direction. This indicates that nano-architecture effects the alignment of the cells, and thus also effects the cells’ morphology, adhesion, and function. It is important to understand the mechanisms of molecular and cellular activities as a response to topographical nano-architecture in order to design a successful neural electrodes (Schulte et al., 2016b). The following sections will describe the proposed mechanism of cellular response to nano-architecture surfaces.

### Role of Proteins: Absorption, Conformation, Integrin Signaling, and Sensing

After a neural electrode is implanted into the brain, ECM proteins immediately aggregate and attach to the electrode surface, thus playing an essential role in determining the duration and stability of the implant (Selvakumaran et al., 2008). It has been shown that topographical cues can modify protein absorption and consequently influence cell interactions via modified receptors, which will lead to changes in mechanotransductive signaling (Selvakumaran et al., 2008). These changes may positively affect cell-implant interactions and ultimately improve implant biocompatibility.

Cell adhesion peptides in the ECM are always the first interaction with an implant (Seidlits et al., 2010). Protein absorption initiates cell adhesion, alignment, and outgrowth of neurites. Yang et al. (2013) observed an increase in protein adsorption onto a nano-structured surface (300 nm wide grooves or pillar gap) compared to a smooth surface, explaining that a possible explanation for the increased protein adsorption may be a result of protein unfolding once adsorbed onto the implant surface, exposing more functional groups for subsequent adhesion of cells. This exposure of amino acids was explored by Webster et al. (2001), who found that when vitronectin is adsorbed onto nano-phase alumina, the protein unfolded, leading to more exposed functional groups that could facilitate cell adhesion and growth. It was proposed by Blattler et al. (2006), that uncontrolled, non-specific interactions between biological molecules and the implant are the reason why implants fail. Blattler et al. (2006) further explained that the modes of failure of implants (i.e., immune reactions), are a result of inadequate protein adsorption onto the implant surface.

**FIGURE 5:**
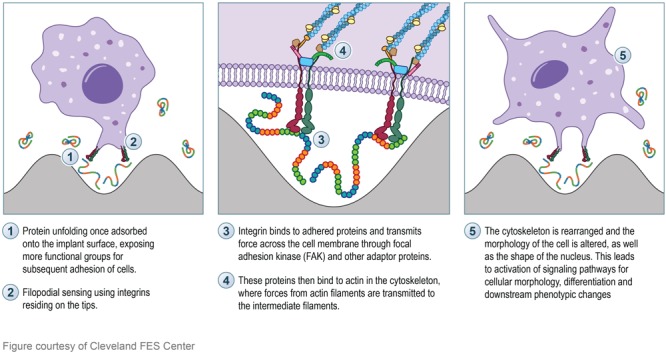
Illustration depicting the proposed mechanism of cell interaction with nano-architecture surface. **Left box:** Protein (rainbow colored strands) adsorption and unfolding onto the nano-architecture surface will initiate subsequent cellular signaling. The unfolding of the protein will expose more functional groups for cell signaling and initiation of molecular pathways. Integrin proteins (red and green) found on the tip of filopodia will sense the protein conformation on the surface. **Middle box:** Integrin binding to adhered surface proteins will transmit force across the cell membrane through FAK (blue rectangle) and other adapter proteins (pink, green, brown, and orange). These proteins then bind to actin in the cytoskeleton (dark blue strands) and transmit force to the intermediate filaments (light blue strands) which send the transmitted force to the cell nucleus. **Right box:** The final morphology of the cell and the cell nucleus are altered, thus effecting the downstream phenotype of the cell.

In addition to protein adsorption on the surface of the implant, integrin proteins residing in a cell’s transmembrane are responsible for the cellular response to changes in the ECM via transmitting force to the cell’s cytoskeleton, thus playing an essential role in cell-substrate binding (Tate et al., 2004; Ingber, 2010). Filopodial probing is crucial for recognition of topographical surface characteristics. Filopodia are thin and only a few micrometers long; they are protrusive processes made by parallel bundles of filamentous actin (Dalby et al., 2004). Molecular receptors such as integrins and cadherins reside on the tips, which behave as sensors for extracellular environments. The length of filopodium outside of the cell membrane is limited to approximately 5 μm, so the range of topographical nano-patterns sensing is limited (Dalby et al., 2004). Albuschies explained that various contact angles between filopodia and substrate result in different performances in sensing of nano-patterned surfaces, which provide guidance to cell alignment (Albuschies and Vogel, 2013). Integrins behave as a mediator of cell adhesion to regulate cellular activities. Integrin binds to ECM proteins in the process of cell recognition, and transmits force across the cell membrane (Tate et al., 2004; Ingber, 2010). Following which adaptor proteins bind to actin in the cytoskeleton, where forces from actin filaments are transmitted to intermediate filaments (Roca-Cusachs et al., 2012). Intermediate filaments are the only cytoskeletal component that have direct access to the filaments in the nucleus (also known as the nucleoskeleton) (Dalby et al., 2007; Eyster and Ma, 2018a). The thought is that the cytoskeletal mechanical stimulation can lead to the rearrangement of interphase chromosomal DNA through the intermediate filaments, thereby effecting gene expression (Bloom et al., 1996; Dalby et al., 2007). Thus, explaining the interactions between cells and their extracellular environment (i.e., nano-architecture implant surface) and the mechanism initiating the signaling pathways for cellular morphology and phenotypic changes (Tate et al., 2004; Roca-Cusachs et al., 2012). **Figure 5** illustrates the aforementioned mechanism of a cell interacting with a nano-architecture surface.

Yang et al. (2013) illustrated that proteins participating in topographical cues can affect signaling of mechanotransduction events by quantitatively analyzing the α5β1 integrin binding to fibronectin-coated nano-patterned substrates with nano-scale shapes, both grooves and pillars. It was found that an increase in focal adhesion correlated with an increased concentration of integrin binding (Yang et al., 2013). Integrin proteins are associated with the strength of the focal adhesions involved in cell-substrate binding, which is directly related to neuron sensitivity to activate signaling pathways of mechanotransduction (Yang et al., 2013; Blumenthal et al., 2014). A later study by Yang et al. (2017) used topography in diverse dimensions to explore focal adhesions and subsequent cell differentiation. Phosphorylated focal adhesion kinase (FAK) gene expression was found to be higher on the smaller nano-scale patterns compared to smooth surfaces (Yang et al., 2017). FAK is a mechanosensitive protein inside the cell and can be activated by integrin binding (Yang et al., 2013). Furthermore, tracking of the α5β1 integrin binding on various substrates showed increased integrin clustering, which was correlated with increased focal adhesion, and contributed to the increase in neuronal density and astrocyte differentiation (Blumenthal et al., 2014). Nano-architecture has also been shown to differentiate cells through mechansotransductive pathways, specifically the mitogen-activated protein kinase/extracellular signal regulated kinase (MEK-ERK) pathway (Yang et al., 2013, 2017). When the MEK/ERK pathway was blocked, there was an observed reduction of downstream signaling from FAK, resulting in a reduction of cell alignment, focal adhesions, neurite outgrowth, and differentiation (Yang et al., 2013). Furthermore, nano-patterned substrates were found to enhance focal adhesions thus leading to neuronal differentiation into dopaminergic and glutamatergic neurons (Yang et al., 2013, 2017).

Changes in cellular activities have a close relationship with protein expression, which can be controlled through nano-architecture surfaces. For example, proteins involved in the regulation of neuronal cytoskeletal organization were upregulated consistently from cells seeded on a nano-structured substrate (Schulte et al., 2016b). Essential proteins involved with axon and synapse microenvironment, as well as vesicle transport and membrane trafficking were also affected by these nano-structured substrates (Schulte et al., 2016b). Maffioli et al. (2017) described that cell–nano-topography interaction can modify cell function such as calcium signaling and/or homeostasis. The effects of surface nano-architecture on cellular functions can be explained by the morphological changes the cells exhibit while on these surfaces (Maffioli et al., 2017).

### Role of Mechanotransduction

Topographical features are important to neural interfacing in terms of local cells, since mechanotransductive components in cells are able to perceive topographical features in the microenvironment. As a result, cells are able to convert mechanical stimuli information to corresponding physiological signals (Altuntas et al., 2016; Schulte et al., 2016a,b; Shi et al., 2016; Bonisoli et al., 2017; Maffioli et al., 2017). Those physiological signals are capable of eliciting further cellular responses and affecting cell function (Bonisoli et al., 2017). Alterations in protein expression are associated with multiple processes: cell–cell adhesion, glycocalyx and ECM, integrin activation and membrane-F-actin linkage, cell–substrate interaction and integrin adhesion complexes, actomyosin organization/cellular mechanics, and nuclear organization and transcriptional regulation (Yang et al., 2015). All of these processes are closely related to mechanotransductive signaling (Yang et al., 2015). It has been exhibited that nano-patterned surfaces, rather than the material itself can cause dramatic change in protein activities (Schulte et al., 2016a).

Despite the lack of clear mechanisms behind cell–surface interactions, there is a clear relationship between mechano-sensitive molecules and the cytoskeleton. Schulte et al. (2016a,b) investigated the mechanism of interfacing of cells and nano-patterned surface by studying supersonic cluster beam deposition (SCBD) of zirconia nanoparticles. Under *in vitro* conditions, a nanoscopic architecture of the adhesion regions was enforced by cell and nano-patterned interfacing, which had an effect on focal adhesion dynamics and the cytoskeletal organization (Schulte et al., 2016b). This also showed that cell morphology was effected by changes in the cytoskeleton structure (Schulte et al., 2016b; Maffioli et al., 2017). This is thought to influence signaling events and cell behaviors because the shape of the cell’s nucleus changes during this process (Ingber, 2010; Maffioli et al., 2017). Furthermore, not only is cell morphology changed, but cell rigidity is also decreased, and mechano-transduction was shown to change transcription factors of neuronal differentiation and protein expression (Schulte et al., 2016b). In fact, it has been shown that activation dynamics of transcription factors was susceptible to mechanical stimuli from topographical features, resulting in cellular protein profile modifications (Schulte et al., 2016a). Several proteins contributing to adhesion and/or cytoskeletal organization lost their functions, which consequently affected neuronal differentiation processes (Schulte et al., 2016a,b).

Living cells usually have a long range of propagation. However, this becomes short-ranged when the actin bundles of the cells are disrupted, or the pre-stress in the actin bundles are inhibited (Wang, 2017). When a force is exerted locally, Src and Rac1 can be directly activated within 300 ms and up to 30–60 μm away (Na et al., 2008; Poh et al., 2009; Wang, 2017). Src (a non-receptor tyrosine kinase protein) and Rac1 (a member of the Rc subfamily within the Rho family of GTP-ases) activation propagates along the plasma membrane along microtubule-dependent mechanisms, causing an elevation of the rigidity of the ECM (Na et al., 2008; Poh et al., 2009; Wang, 2017). The Rho/Rac GTPase signaling pathway is particularly important in cell/nano-feature mechanism studies (Eyster and Ma, 2018a). RhoA has been shown to play a role with controlling cell adhesion (Kaibuchi et al., 1999), cell spreading (Thodeti et al., 2003), cytoskeletal stress fiber formation (Buhl et al., 1995), as well as stem cell differentiation (McBeath et al., 2004). Moreover, mechanotransductive signaling through the cytoplasm is extremely fast, about 40 times faster than chemosignaling, as a result of the pre-stressed fibers which are stiffer than the rest of the cytoplasm, allowing stresses and stimuli to travel along the whole length of the cell quickly (Na et al., 2008; Poh et al., 2009; Wang, 2017). The ability for fast mechanotransductive signaling compared to chemo-signaling, even across long distances such as 30–50 μm, may contribute to the importance of surface architecture on the cellular morphology of cells and why architecture may even be more important than surface chemistry.

### Role of Nano-Architecture on Cell Phenotype and Differentiation

The addition of nano-architecture on surfaces has been implicated to alter the phenotype of cells as well as control cell differentiation. Additionally, nano-architecture topographical features can provide guidance to neuronal extension, such as the direction and length of neuron growth, which promote neuronal regeneration (Altuntas et al., 2016; Schulte et al., 2016a; Shi et al., 2016; Bonisoli et al., 2017; Maffioli et al., 2017; Yang et al., 2017). Maffioli et al. (2017) explored mechanosensing/transduction and cell differentiation with multiple surface topographies. By quantitatively analyzing phosphoproteomic data of diverse topographical profiles, it was observed that the dynamic and complex modulation of the entire signaling network was affected by the cell’s interaction with nano-structures, which contributes to distinct cellular behaviors (Maffioli et al., 2017). Schulte et al. (2016a) reported that nano-topographical features that mimic ECM could control the level of maturation of neural networks. By observing neuron morphology with atomic force microscopy, Schulte et al. (2016a) found that neurons grown on nano-patterned surfaces expanded more neurites and accelerate synaptogenesis, which significantly regulates neuronal differentiation and maturation.

Conductive polymers with electrical conductive properties that can give electrical stimulus are commonly used biomaterials involved in nano-patterned topography studies, which is favorable for neuronal differentiation (Ingber, 2010; Altuntas et al., 2016; Shi et al., 2016; Bonisoli et al., 2017). Bonisoli et al. (2017) combined topographical features with a conductive polymer coating to support neuronal growth and differentiation, and axonal guidance. The device conveyed multiple stimuli, both mechanotransductively and electrically, to cells and brain tissues, which effectively promoted neurite growth (Bonisoli et al., 2017). Yang et al. (2017) seeded human neural stem cells (hNSCs) over nano-patterned titanium substrates. They found that cells seeded on the nano-patterned substrate showed alignment and significant focal adhesions, which led to enhanced neuronal differentiation (Yang et al., 2017). Altuntas et al. (2016) extended the application of nano-porous anodized alumina membranes (AAMs) to neural implant coatings. The conductive property of film was achieved by coating AAMs with a thin conducting layer (CAAMs). The conductive AAMs showed that they were favorable for neurite extension and proliferation under electrical stimulation but poor cell adhesion performance (Altuntas et al., 2016). The nano-porous featured AAMs without conducting layer gave topographical cues and thus had excellent neuronal cell adhesion (Altuntas et al., 2016). With nerve growth factor embedded, naked AAMs could achieve similar effect of neurite extension compared to electrically stimulated CAAMs (Altuntas et al., 2016).

While the topographical surface of neural implants provides mechano-transductive cues so that absorbed proteins are modified, the aggregated cells also change their morphology due to local environmental changes (Szarowski et al., 2003). Attachment and clustering of microglia and astrocytes on the implant surface is common in *in vivo* studies (Szarowski et al., 2003). The additional mechano-tranductive signaling of topography is of great importance in cell–protein interactions, which further changes the cell phenotype and has effects on neuro-inflammation (Szarowski et al., 2003). Neuronal cell differentiation is also known to be affected by topography. McMurray et al. (2011) was able to design a nano-patterned substrate that could control differentiation of mesenchymal stem cells. However, unlike other groups where speeding up or specifying a certain type of cell differentiation was investigated, McMurray et al. (2011) tested the ability of a certain nano-structure to delay differentiation. They were able to identify a nano-structure surface (120 nm pits in a square configuration spaced 300 nm apart, with an offset level near zero) able to delay differentiation of stem cells to remain in their undifferentiated phenotype for over 8 weeks post-seeding (McMurray et al., 2011). In this *in vitro* investigation of adult stem cells differentiation, the nano-structured surface promoted the effect of small RNAs correlated with cell signaling and metabolomics, which manipulated the long-term differentiation of mesenchymal stem cells (McMurray et al., 2011). Christopherson et al. (2009) demonstrated the effect of nano-scale topographical cues on neuronal differentiation and outgrowth processing of human stem cells. The dimensions of nano-patterned geometries enhanced the alignment of neural process outgrowth with the direction of the nano-patterns (Christopherson et al., 2009). The increase in neuronal alignment and outgrowth processing significantly promotes compatibility with implanted devices (Christopherson et al., 2009). Yoo et al. (2015) elucidated that nano-grooved patterns made via UV-lithography were able to enhance fibroblast differentiation into dopaminergic neurons, concluding that nano-pattern substrates could serve as an efficient stimuli for cell differentiation. Collectively, the examples provided here utilizing architecture in the nano-scale, give evidence that nano-architecture have an effect on the phenotype, morphology, and differentiation of cells.

## Conclusion and Future Perspectives

Integration of neural electrodes into the brain tissue lies heavily on reducing the neuroinflammatory response. An understanding of the natural environment and how cells interact and communicate with each other is crucial when designing next generation neural electrodes. A growing body of literature is investigating the effect surface architectures have on controlling cell behavior, differentiation, and phenotype. Here, we reviewed only the nano-scale architectures, as nano-scale surface modifications have shown promise in controlling protein adsorption, reducing glial cell inflammatory markers, guiding axonal direction, and cell differentiation. Nano-scale architectures are inspired by the native *in vivo* environment, specifically the ECM which cells receive their signals and cues from. Advancements in fabrication techniques and novel biomaterials have allowed the addition of nano-scale features to be added to neural implants within the manufacturing process or even post-processing. For example, altering the architecture of commercially used microelectrodes, such as the Michigan electrode, utilizing FIB lithography, allows seamless translation to research labs and potentially patients. Additionally, the fabrication processes to create nano-architectures can be done on numerous materials. The feature sizes and shapes can be limitless with the multitude of fabrication methods. Nano-architectures can include multiple features on various parts of the neural electrodes depending on the desired outcome. Features can be integrated onto the contact sites to reduce impedance and increase recording quality as well as around the electrode insulating layer to reduce the foreign body response. The nano-scale features on the electrode’s insulating layer help to reduce the foreign body response by controlling the protein adsorption, conformation, integrin signaling thereby influencing the cellular morphology, and downstream phenotype. The promise of incorporating nano-architechture on the surface of neural implants has been implicated by the countless aforementioned examples. Nano-architecture can be utilized to control cell phenotype, differentiation, growth, adhesion, migration, and morphology.

This approach can be utilized on various neural implants, including stimulating electrodes, DBS probes, closed loop sensors. Unfortunately, there is a lack of preclinical studies evaluating abovementioned *in vitro* studies presented throughout this review. Given that the methods to incorporate nano-architecture onto neural electrodes are feasible, we predict the gap in the literature evaluating this approach will be filled in the upcoming years. Although a proposed mechanism of how nano-architectures can control cellular response was reviewed, it is crucial to consider diverse features will elicit different cellular responses. Thus, a thorough understanding of cellular responses to specific nano-architectures will inform the design and development of improved neural prosthetic and neuromodulatory devices. The implementation of nano-architectures onto these devices is hypothesized to reduce the foreign body response and create seamless device tissue integration. The ability for nano-architectured implants to more closely mimic the brain’s architecture is an interesting avenue of discovery and research, because it allows for the future development of the optimal implant that integrates seamlessly with CNS tissue.

## Author Contributions

YK and EE contributed substantially to the conception and design of the work, drafting and revising the manuscript for important intellectual content, approved the final version to be published, and agree to be accountable for all aspects of the work. SM, KC, HF, JR, and AH-D drafted corresponding sections of the manuscript. All authors (EE, YK, SM, KC, HF, JR, and A-HD) approved the final version to be published and agree to be accountable for all aspects of the work.

## Conflict of Interest Statement

The authors declare that the research was conducted in the absence of any commercial or financial relationships that could be construed as a potential conflict of interest.
